# Molecular and morphological characterisation of *Pharyngostrongylus kappa* Mawson, 1965 (Nematoda: Strongylida) from Australian macropodid marsupials with the description of a new species, *P. patriciae* n. sp.

**DOI:** 10.1186/s13071-018-2816-6

**Published:** 2018-04-27

**Authors:** Tanapan Sukee, Ian Beveridge, Abdul Jabbar

**Affiliations:** Department of Veterinary Biosciences, Melbourne Veterinary School, Faculty of Veterinary and Agricultural Sciences, The University of Melbourne, Werribee, Victoria 3030 Australia

**Keywords:** *Pharyngostrongylus*, Cloacininae, Macropodid marsupials, Internal transcribed spacers, Phylogenetics, Morphology

## Abstract

**Background:**

*Pharyngostrongylus kappa* Mawson, 1965 is a nematode (Strongyloidea: Cloacininae), endemic to the sacculated forestomachs of Australian macropodid marsupials (kangaroos and wallaroos). A recent study revealed genetic variation within the internal transcribed spacer region of the nuclear ribosomal DNA among *P. kappa* specimens collected from *Macropus giganteus* Shaw and *Osphranter robustus* (Gould). This study aimed to characterise the genetic and morphological diversity within *P. kappa* from four macropodid host species, including *M. giganteus*, *O. robustus*, *O. antilopinus* (Gould) and *O. bernardus* (Rothschild).

**Methods:**

Specimens of *P. kappa* from *M. giganteus* and *Osphranter* spp. from various localities across Australia were examined. The first and second internal transcribed spacers (ITS1 and ITS2, respectively) were amplified using polymerase chain reaction and sequenced. Phylogenetic methods were used to determine the interspecific diversification within *P. kappa* and its evolutionary relationship with other congeners.

**Results:**

Morphological examination revealed that *P. kappa* from *M. giganteus*, the type-host, can be distinguished from those in *Osphranter* spp. by the greater length and number of striations on the buccal capsules. DNA sequences showed that *P. kappa* from *M. giganteus* was genetically distinct from that in *Osphranter* spp., thereby supporting the morphological findings. Based on these finding, a new species from *Osphranter* spp., *Pharyngostrongylus patriciae* n. sp., is described.

**Conclusion:**

*Pharyngostrongylus patriciae* n. sp. from *Osphranter* spp. is distinguished from *P. kappa* based on molecular and morphological evidence. The study highlights the importance of combining molecular and morphological techniques for advancing the nematode taxonomy. Although ITS genetic markers have proven to be effective for molecular prospecting as claimed in previous studies, future utilisation of mitochondrial DNA to validate ITS data could further elucidate the extent of speciation among macropodid nematodes.

**Electronic supplementary material:**

The online version of this article (10.1186/s13071-018-2816-6) contains supplementary material, which is available to authorized users.

## Background

Kangaroos and wallabies (family Macropodidae) are an important component of the Australian endemic fauna [1], and owing to their grazing habits, they harbour an abundant and extensive range of strongyloid helminths in their gastrointestinal tracts [2, 3]. Macropodid helminths are highly host specific [4], with at least 372 species described so far [3, 5–7]. The primary focus of the research into macropodid helminths has been quantifying biodiversity and assessing the prevalence and distribution of these parasites [3]. Unlike helminths of livestock, pathogenic effects, host-parasite interactions and life-cycle stages of the helminth fauna of macropodid marsupials are not well understood [3, 8]. Nonetheless, current knowledge suggest that the most outstanding feature observed in helminths from macropodid hosts is their remarkable diversity, particularly among the nematodes [2, 3, 6, 9].

The subfamily Cloacininae Stossich, 1899 (Nematoda: Strongylida) comprising 36 genera and 256 species, is one of the most morphologically diverse groups of mammalian parasites [3]. All members of the Cloacininae occur exclusively in the oesophagus and forestomach of macropodid and the potoroid marsupials. York & Maplestone [10] established *Pharyngostrongylus* Yorke & Malplestone, 1926 for a single nematode species characterised by transverse striations on the buccal capsule, *Pharyngostrongylus macropodis* Yorke & Malplestone, 1926 found in the stomach of the agile wallaby *Notamacropus agilis* (Gould). Subsequently, additional species with these characteristics were discovered in the stomachs of other macropodid hosts [11]. Presently, 14 species of *Pharyngostrongylus* are known, with *P. thylogale* Chilton, Huby-Chilton, Gasser, Koehler & Beveridge, 2016 the most recent addition to the genus [12].

*Pharyngostrongylus kappa* Mawson, 1965 occurs in the eastern grey kangaroo, *Macropus giganteus* Shaw, throughout its entire distribution along the eastern coast of Australia. However, it also occurs in the common wallaroo, *Osphranter robustus* (Gould), the antilopine wallaroo, *O. antilopinus* (Gould), and the black wallaroo, *O. bernardus* (Rothschild), in the northern parts of Australia. *Pharyngostrongylus kappa* is morphologically distinguished from other congeners by the presence of an elongated buccal capsule with transverse striations and eight petaloid labial crown elements surrounding the apical opening [11]. A recent study of the phylogeny of the ITS region within *Pharyngostrongylus* found that three specimens of *P. kappa* obtained from *M. giganteus* in Victoria and *O. robustus* from Queensland and the Northern Territory were genetically distinct from one another [12]. These data suggest the existence of genetic variation within *P. kappa*. Given that occurrences of distinct genetic species in different hosts have been observed in many species within the subfamily Cloacininae [13–17], it was hypothesised that *P. kappa* from different macropodid hosts and geographical localities might exhibit genetic and possibly morphological variation. Therefore, this study aimed to characterise the genetic and morphological variation among *P. kappa* from the different macropodid hosts and localities within Australia. The study included the remaining species of *Pharyngostrongylus* known from wallaroos, namely *P. papillatus* Beveridge, 1982 and *P. sharmani* Beveridge, 1982.

## Methods

### Specimen collection

**Fig. 1:**
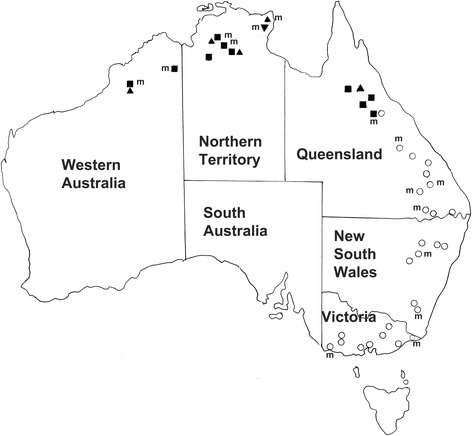
Distribution of *Pharyngostrongylus kappa* ex *Macropus giganteus* (open circles) and *P. patriciae* n. sp. ex *Osphranter robustus* (closed squares), *O. antilopinus* (closed triangles) and *O. bernardus* (inverted triangle). The symbol ‘m’ indicates localities from which material included in molecular studies was collected

Adult specimens of *Pharyngostrongylus* spp. (*n* = 173) used in this study were made available from the frozen parasite collection at the Veterinary School of The University of Melbourne. These worms had been collected from the stomachs of culled or road-killed macropodid marsupials, including eastern grey kangaroos (*M. giganteus*), common wallaroos (*O. robustus*), antilopine wallaroos (*O. antilopinus*) and black wallaroos (*O. bernardus*) (Fig. 1; Table 1).

**Table 1:** *Pharyngostrongylus* spp.: specimens used for obtaining molecular data in this study with details of their hosts, localities, and deposition of morphological voucher specimens

Parasite	Host	Locality	Coordinates	No. of specimens examined	SAM registration number
*P. kappa*	*M. giganteus*	Taroom, Qld	25°38′S, 149°47′E	10	
*P. kappa*	*M. giganteus*	Inglewood, Qld	28°24′S, 151°4′E	11	48133
*P. kappa*	*M. giganteus*	Mungallala, Qld	26°26′S, 147°32′E	8	
*P. kappa*	*M. giganteus*	35 km South Clermont, Qld	22°49′S, 147°37′E	11	48136
*P. kappa*	*M. giganteus*	Bondo State Forest, NSW	35°17′S, 148°34′E	7	48132
*P. kappa*	*M. giganteus*	Coonabarabran, NSW	31°16′S, 149°13′E	8	48137
*P. kappa*	*M. giganteus*	Portland, Vic	38°21′S, 141°36′E	12	48134
*P. kappa*	*M. giganteus*	Genoa, Vic	37°27′S, 149°41′E	4	48135
*P. patriciae*	*O. antilopinus*	87 km SW of Katherine, NT	14°27′S, 132°16′E	3	
*P. patriciae*	*O. antilopinus*	70 km S of Maningrida, NT	12°3′S, 134°13′E	1	48142
*P. patriciae*	*O. antilopinus*	Napier Downs Station, WA	17°19′S, 124°48′E	5	
*P. patriciae*	*O. bernardus*	80 km S of Maningrida, NT	12°3′S, 134°13′E	17	48141
*P. patriciae*	*O. robustus*	32 km SW of Katherine, NT	14°27′S, 132°16′E	2	
*P. patriciae*	*O. robustus*	Mabel Downs Station, WA	17°21′S, 128°1′E	4	48138
*P. patriciae*	*O. robustus*	Edith River, NT	14°51′S, 131°53′E	9	48139
*P. patriciae*	*O. robustus*	Charters Towers, Qld	25°38′S, 149°47′E	13	48140
*P. papillatus*	*O. antilopinus*	87 km SW of Katherine, NT	14°27′S, 132°16′E	1	
*P. papillatus*	*O. bernardus*	80 km S of Maningrida, NT	12°3′S, 134°13′E	12	48144
*P. papillatus*	*O. robustus*	32 km SW of Katherine, NT	14°27′S, 132°16′E	10	
*P. papillatus*	*O. robustus*	Mabel Downs Station, WA	17°21′S, 128°1′E	8	48145
*P. papillatus*	*O. robustus*	Edith River, NT	14°51′S, 131°53′E	4	
*P. sharmani*	*O. antilopinus*	87 km SW of Katherine, NT	14°27′S, 132°16′E	3	
*P. sharmani*	*O. antilopinus*	Napier Downs Station, WA	17°19′S, 124°48′E	3	48143
*P. sharmani*	*O. bernardus*	80 km S of Maningrida, NT	12°3′S, 134°13′E	6	
*P. sharmani*	*O. robustus*	Edith River, NT	14°51′S, 131°53′E	1	

*Abbreviations*: *NSW* New South Wales, *NT* the Northern Territory, *Qld* Queensland, *Vic* Victoria, *WA* Western Australia, *SAM* South Australian Museum, Adelaide

### Morphological characterisation

Prior to examination, the frozen specimens (at -80 °C) were thawed and individual worms were cut into three pieces. The anterior and posterior ends were cleared in lactophenol, mounted on slides and three morphologically distinct species of *Pharyngostrongylus* (*P. kappa*, *P. papillatus* and *P. sharmani*) were identified (Table 1). The mid-body portion of each worm was placed in an Eppendorf tube with 0.5 ml of H_2_O, labelled and frozen at -80 °C until required for molecular analysis.

Additionally, specimens of *P. kappa* in the collections of the South Australian Museum (SAM), Adelaide were also examined (see Additional file 1: Table S1). Ten males and ten female worms from different hosts and localities were selected for morphological measurements where available. The selected nematodes were cleared in lactophenol, mounted on slides and measurements were made using an ocular micrometer. Measurements are presented as the range and the mean in millimetres. Morphological drawings were made with the aid of a drawing tube attached to the Olympus BH-2/BHS Systems microscope. Photographs of the buccal capsules were taken with an attached microscope digital camera, Olympus DP21. The terminology used in the morphological description follows Beveridge [11] while nomenclature of hosts and parasites species follows Spratt & Beveridge [18].

Discriminant function analyses (DFA) were performed separately on male (*n* = 44) and female (*n* = 50) measurements using Microsoft Excel with XLSTAT [19]. Predictor variables included length, oesophageal length, buccal capsule length, buccal capsule width and spicule length for males. The same predictor variables were used in analysis for the females but included tail length and distance from the vulva to the posterior extremity instead of spicule length.

### Molecular characterisation

To detect intraspecific genetic variation among specimens of *P. kappa* from different hosts and geographical localities, the first and second internal transcribed spacers (ITS1 and ITS2, respectively) were amplified using polymerase chain reaction (PCR). The ITS regions of *P. papillatus* and *P. sharmani* were also included in the molecular analyses to determine the phylogenetic relationship of *P. kappa* to other congeners.

Prior to DNA extraction, frozen mid-body segments of individual nematodes were thawed and rinsed three times in H_2_O. Genomic DNA was extracted using minicolumns (Wizard® SV Genomic DNA purification Kit, Promega, Madison, WI, USA) according to the manufacturer’s recommendations. The concentration and purity of each extracted DNA sample were determined using a NanoDrop spectrophotometer, and then stored in Eppendorf tubes at 4°C until required.

The ITS1 and ITS2 from the nuclear ribosomal DNA were amplified from 2 μl of each DNA sample using PCR. The primers used were NC16 (5'-AGT TCA ATC GCA ATG GCT T- 3')/NC13R (5'-GCT GCG TTC TTC ATC GAT-3') and NC1 (5'-ACG TCT GGT TCA GGG TTG TT-3')/NC2 (5'-TTA GTT TCT TTT CCT CCG CT-3') for the ITS1 and ITS2, respectively [20]. PCRs were conducted in 25 μl volumes containing 10 mM Tris-HCl (pH 8.4), 50 mM KCl (Promega), 3.5 mM MgCl2, 250 μm of each deoxynucleotide triphosphate (dNTP), 25 pmol of each primer and 1 U of GoTaq polymerase (Promega). PCR cycling conditions were: 94 °C for 5 min, then 35 cycles of 94 °C for 30 s, 55 °C for 20 s, and 72 °C for 20 s, followed by 72 °C for 5 min. Negative (no DNA) and positive controls [*Haemonchus contortus* (Rudolphi, 1803) DNA] were included in each PCR run. Following PCR, aliquots (5 μl) of individual amplicons were examined by agarose gel electrophoresis [1.5% gels in 0.5 TAE buffer (20 mM Tris, 10 mM acetic acid, 0.5 mM EDTA)]. Gels were stained using GelRed Nucleic Acid Gel Stain (Biotium GelRed stain, Fisher Scientific, Waltham, Massachusetts, USA), subjected to transillumination and photographed using a gel documentation system (Kodak Gel Logic 1500 Imaging System, Eastman Kodak Company, Rochester, NY, USA).

For each spacer region, amplicons were treated with shrimp alkaline phosphatase and exonuclease I [21] and then subjected to automated DNA sequencing using the 96-capillary 3730xl DNA Analyser, (Applied Biosystems, Foster City, CA, USA) at Macrogen Inc., South Korea. Sequencing of ITS1 and ITS2 was conducted using the specific PCR primers in separate reactions. The quality of nucleotide sequences was appraised using the program Geneious R10 (Biomatters Ltd., Auckland, New Zealand) [22], and polymorphic sites were designated using International Union of Pure and Applied Chemistry (IUPAC) codes. Voucher morphological specimens representing each of the genotypes found in the analysis were deposited in SAM (Table 1).

### Phylogenetic analysis

The ITS1 and ITS2 sequences of each individual nematode generated in this study were compared with those of *P. kappa* previously published from *M. giganteus* (Genoa, Vic; GenBank: LT576294) and *O. robustus* (Charters Towers, Qld and Edith River, NT; GenBank: LT576296 and LT576295) [12]. Prior to phylogenetic analysis, the sequences were aligned using the program MEGA 7.0.26 [23] by employing multiple sequence comparison by log-expectation (MUSCLE) [24]. The alignments were then adjusted manually using the program BioEdit [25]. Pairwise comparisons to determine the relatedness among sequences were conducted using MEGA.

Phylogenetic analyses were performed on individual (ITS1 or ITS2) or concatenated ITS1 and ITS2 (designated as ITS+) sequence datasets using the Neighbour-Joining (NJ) and Bayesian Inference (BI) methods. Based on the sequence data, the most suitable likelihood parameters and evolutionary models were determined using the Akaike information criteria test in the program jModeltest version 3.7 [26]. jModeltest revealed that the most suitable model for the ITS1, ITS2 and ITS+ were TPM1uf+I, TPM3uf and GTR+I+G, respectively. The BI analysis was conducted, employing the Markov Chain Monte Carlo (MCMC) method in MrBayes 3.1.2 [27, 28]. Posterior probabilities were calculated using 2,000,000 generations, employing four simultaneous tree-building chains, with every 100th tree being saved. A consensus tree (50% majority rule) was constructed based upon the remaining trees generated by BI. The NJ analyses were performed employing MEGA and the nodes were tested for robustness with 10,000 bootstrap replicates. The ITS sequences of *Cloacina ernabella* Johnston & Mawson, 1938 were used as the outgroup. The topology of each tree was examined for concordance between the NJ and BI analysis in the program FigTree.

## Results

Of the 173 adult *Pharyngostrongylus* specimens examined, *P. kappa* was identified from four macropodid host species, including *M. giganteus* (*n* = 71; Qld, NSW and Vic); *O. robustus* (*n* = 28; NT and Qld), *O. bernardus* (*n* = 17; NT) and *O. antilopinus* (*n* = 9; NT and WA) (Table 1). *Pharyngostrongylus papillatus* was identified from *O. robustus* (*n* = 22; WA), *O. antilopinus* (*n* = 1; NT) and *O. bernardus* (*n* = 12; NT)*. Pharyngostrongylus sharmani* was identified from *O. bernardus* (*n* = 6; NT), *O. robustus* (*n* = 1; WA) and *O. antilopinus* (*n* = 6; WA) (Table 1).

Male and female nematodes from the different macropodid hosts were similar in length and width (Table 2). The average lengths of the specimens from *M. giganteus* were slightly greater than those of the specimens from *Osphranter* spp. (male: 8.25 *vs* 5.45–7.46 mm; female: 9.52 *vs* 6.90–9.30 mm). Other morphological features such as the distances of the nerve-ring, deirids and excretory pore to the anterior extremity fell within the same range in specimens examined from all hosts. Lengths of the male spicules also overlapped in specimens from each host. However, the length of the buccal capsule of *P. kappa* from *M. giganteus* was greater (0.12–0.17 mm) than from *Osphranter* spp. (0.08–0.13 mm) (Table 2). Counting of the number of transverse striations on buccal capsules revealed that the specimens from *M. giganteus* had 60–64 striations, whereas those from *Osphranter* spp. had 38–50 striations (Table 2).

**Table 2:** Measurements in millimetres of morphological features of male and female specimens of *Pharyngostrongylus kappa* and *Pharyngostrongylus patriciae* from different host species

Host species	*Pharyngostrongylus kappa*		*Pharyngostrongylus patriciae* n. sp.					
	*M. giganteus* (*n* = 10)		*O. robustus* (*n* = 10)		*O. antilopinus* (*n* = 5)		*O. bernardus* (*n* = 10)	
	Range	Mean	Range	Mean	Range	Mean	Range	Mean
Males	(*n* = 10)		(*n* = 10)		(*n* = 5)		(*n* = 10)	
TL	7.10–9.60	8.25	5.85–8.60	7.46	5.95–8.20	7.27	5.0–6.05	5.45
MW	0.32–0.47	0.37	0.23–0.46	0.34	0.21–0.33	0.28	0.16–0.28	0.21
BC	0.13–0.17	0.15	0.09–0.13	0.11	0.08–0.12	0.11	0.09–0.10	0.09
BCW	0.02–0.03	0.03	0.02–0.03	0.02	0.03–0.03	0.03	0.02–0.02	0.02
BCLW	5.60–6.80	5.68	3.33–6.50	3.98	3.20–4.80	4.24	4.25–6.33	5.09
OE	1.64–1.87	1.74	1.32–1.93	1.57	1.38–1.80	1.52	1.36–1.63	1.48
NR	0.31–0.38	0.35	0.26–0.38	0.32	0.30–0.36	0.36	0.25–0.30	0.28
DD	0.09–0.15	0.12	0.08–0.14	0.11	0.06–0.12	0.10	0.07–0.11	0.09
EP	0.44–0.61	0.52	0.27–0.50	0.41	0.41–0.57	0.50	0.35–0.43	0.37
SP	2.30–2.68	2.49	2.00–2.46	2.18	2.05–2.30	2.17	1.62–2.35	1.90
Females (*n* = 10)								
TL	6.70–12.7	9.52	8.05–10.35	9.30	8.50–9.10	8.79	5.45–8.90	6.90
MW	0.35–0.57	0.46	0.31–0.50	0.40	0.28–0.35	0.32	0.23–0.38	0.3
BC	0.12–0.17	0.14	0.09–0.11	0.11	0.09–0.09	0.09	0.09–0.11	0.11
BCW	0.03–0.04	0.03	0.02–0.03	0.02	0.03–0.03	0.03	0.02–0.03	0.02
BCLW	4.67–6.80	5.68	3.17–5.20	3.86	3.40–5.20	4.34	4.00–6.70	5.13
OE	1.54–2.23	1.90	1.50–2.17	1.85	1.52–2.01	1.73	1.40–2.20	1.76
NR	0.3–0.46	0.36	0.30–0.41	0.34	0.28–0.35	0.32	0.26–0.32	0.30
DD	0.11–0.15	0.12	0.08–0.14	0.10	0.07–0.10	0.10	0.08–0.12	0.11
EP	0.06–0.61	0.46	0.38–0.56	0.47	0.42–0.56	0.48	0.35–0.51	0.41
TA	0.43–0.68	0.51	0.34–0.47	0.38	0.35–0.45	0.40	0.24–0.41	0.35
VU	0.68–1.05	0.85	0.65–0.78	0.68	0.65–0.77	0.70	0.50–0.71	0.70
VG	0.85–1.35	1.04	0.70–0.98	0.82	0.50–0.75	0.67	0.50–0.73	0.62

*Abbreviations*: *TL* total length, *MW* maximum width, *BC* buccal capsule length, *BCW* buccal capsule width, *BCLW* buccal capsule length to width ratio, *OE* length of oesophagus, *NR* distance from anterior end of nerve-ring to anterior extremity, *DD* distance from deirid to anterior extremity, *EP* distance from excretory pore to anterior extremity, *SP* spicule length, *TA* female tail length, *VP* distance from vulva to posterior extremity, *VA* length of vagina

**Fig. 2:**
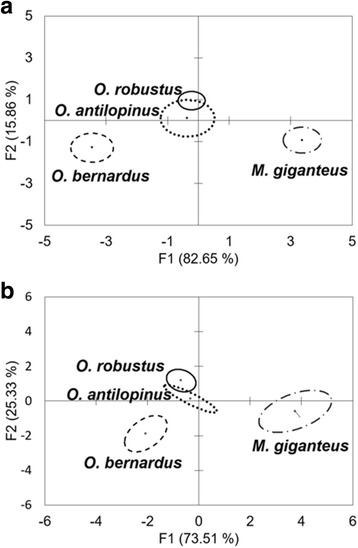
Differentiation of male (**a**) and female (**b**) *Pharyngostrongylus kappa* specimens based on discriminant functions 1 (F1) and 2 (F2). Centroids are shown as small circles within each host group

The discriminant analyses showed that the first two functions (i.e. F1 and F2) combined were able to correctly assign 98.51% of the male specimens (Fig. 2a) and 98.84% of female specimens (Fig. 2b). In both sexes, there were slight overlaps between specimens from *O. robustus* and *O. antilopinus* whilst those from *M. giganteus* and *O. bernardus* each grouped separately.

- **Order Strongylida Railliet & Henry, 1913**
- **Family Charbetiidae Popova, 1955**
- **Subfamily Cloacininae Stossich, 1899**
- **Genus** ***Pharyngostrongylus*** **York & Maplestone, 1926**

### *Pharyngostrongylus patriciae* n. sp.

***Type-host*:** *Osphranter robustus woodwardi* (Thomas) (Marsupialia: Macropodidae).

***Other hosts:*** *Osphranter antilopinus* (Gould) (Marsupialia: Macropodidae)*, Osphranter bernardus* (Rothschild) (Marsupialia: Macropodidae)*.*

***Type-locality*:** Pine Creek (13.82°S, 131.83°E) Northern Territory, Australia.

***Other localities*:** Specimens from *Osphranter robustus* were collected from Edith River (Northern Territory), Katherine (Northern Territory), south west of Katherine (Northern Territory), Willeroo Station, Katherine (Northern Territory), Newry Station, Timber Creek (Northern Territory), 5 km north of Mataranka (Northern Territory), 24 km east of Georgetown (Queensland), Bluewater Springs (Queensland), 30 km north of Charters Towers (Queensland), Lyndhurst (Queensland), Mabel Downs Station, Kununurra (Western Australia) and Napier Range (Western Australia), Australia. Specimens from *Osphranter antilopinus* were collected from 87 km south-west of Katherine (Northern Territory), 70 km south of Maningrida (Northern Territory), Burlington Station, Mount Surprise (Queensland), 20 km east of Mount Surprise (Queensland) and Napier Downs Station, Derby (Western Australia), Australia. Specimens from *Osphranter bernardus* were collected from 80 km south of Maningrida (Northern Territory), Australia.

***Type-material*:** Holotype, male from *O. r. woodwardi* (SAM 48125); allotype female (SAM 48126); paratypes 30 males and 52 females, same data (SAM 48127).

***Voucher material*:** From *Osphranter robustus*: Northern Territory: 3 ♂♂, 3 ♀♀, Katherine (SAM 25763); 3 ♂♂, 4 ♀♀, 32 km south west of Katherine (SAM 23697); 2 ♀♀, Willeroo Station, Katherine (SAM 48124); 11 ♂♂, 22 ♀♀, Edith River (SAM 32723); 1 ♂ 2 ♀♀, Newry Station, Timber Creek (SAM 48122); 5 ♂♂, 6 ♀♀, 5 km north of Mataranka (SAM 48123); Queensland: 2 ♂♂, 2 ♀♀, 24 km east of Georgetown (SAM 25415); 2 ♂♂, 4 ♀♀, Bluewater Springs (SAM 25417);3 ♂♂, 4 ♀♀, 30 km north of Charters Towers (SAM 32487); 4 ♂♂, 21 ♀♀, Lyndhurst (SAM 32745); Western Australia: 6 ♂♂, 8 ♀♀, Mabel Downs Station, Kununurra (SAM 45975); 1 ♂, Napier Range (SAM 23043). From *Osphranter antilopinus*: Northern Territory: 4 ♂♂, 16 ♀♀, 87 km south-west of Katherine (SAM 32712); 2 ♀♀, 70 km south of Maningrida (SAM 32797); Queensland: 1 ♂, 1 ♀, Burlington Station, Mount Surprise (SAM 6461); 1 ♂, 6 ♀♀, 20 km east of Mount Surprise (SAM 25936); Western Australia: 5 ♂♂, 12 ♀♀, Napier Downs Station, Derby (SAM 45972). From *Osphranter bernardus*: Northern Territory: 32 ♂♂, 53 ♀♀, 80 km south of Maningrida (SAM 32803).

***Comparative material studied*:** *P. kappa* from *Macropus giganteus*: Queensland: 3 ♂♂, 5 ♀♀, Townsville (SAM 7279); 12 ♂♂, 18 ♀♀, Hervey’s Range, Townsville (SAM 7677); 17 ♂♂, 10 ♀♀, Woodstock (SAM 7353); 6 ♂♂, 4 ♀♀, Charters Towers (SAM 7397, 24246); 1♀, Warrawee Station via Charters Towers (SAM 12300); 6 ♂♂, 15 ♀♀, Harvest Home Station via Charters Towers (SAM 13380, 13495, 13496); 1♀, Clermont (SAM 24275); 15 ♂♂, 13 ♀♀, Rockhampton (SAM 11062); 2 ♂♂, 7 ♀♀, Melmoth Station via Dingo (SAM 19901); 1 ♂, 5 ♀♀, Darling Plains Station via Banana (SAM 19902); 2 ♂♂, 2 ♀♀, Theodore (SAM 23230); 21 ♂♂, 11 ♀♀, Mungallalla (SAM 23234); 6 ♂♂, 8 ♀♀ Bogantungan (SAM 24254); 2 ♂♂, 2 ♀♀, Moonie (SAM 25698); 14 ♂♂, 13 ♀♀, Inglewood (SAM 23223); 8 ♂♂, 8 ♀♀ Killarney (SAM 19907); New South Wales: 5♂♂, 12 ♀♀, Armidale (SAM 8649); 15 ♂♂, 4 ♀♀, Kingstown (SAM 10606); 2 ♂♂, 1♀, Pilliga (SAM 19599); 19 ♂♂, 10 ♀♀, Coonabarabran (SAM 23241); 2 ♀♀, Gilgandra (SAM 24691); 22 ♂♂, 13 ♀♀, Bondo State Forest (SAM 19899); Australian Capital Territory: 12 ♂♂, 14♀♀, Tidbinbilla (SAM10939); Victoria: 8 ♂♂, 17 ♀♀, Dartmouth (SAM 9215); 6 ♂♂, 3 ♀♀, Zumsteins (SAM 9275); 13 ♂♂, 8 ♀♀, Yan Yean (SAM 9620); 14 ♂♂, 13 ♀♀, Marlo Plains (SAM 9709); 1 ♂, 1 ♀, Mirranatwa (SAM 10904); 1 ♀, Fraser National Park (SAM 11047); 2 ♂♂, 3 ♀♀, Lara (SAM 34620); 7 ♂♂, 4 ♀♀, Portland (SAM 31551).

***Site in host*:** Stomach

***Representative DNA sequences*:** The first and second internal transcribed spacers sequences were deposited in the GenBank database under the accession numbers MG972159 and MG972159.

***ZooBank registration*:** To comply with the regulations set out in article 8.5 of the amended 2012 version of the *International Code of Zoological Nomenclature* (ICZN) [29], details of the new species have been submitted to ZooBank. The Life Science Identifier (LSID) of the article is urn:lsid:zoobank.org:pub:11FD1405-58DB-4C37-BA00-4044F12FEAC1. The LSID for the new name *Pharyngostrongylus patriciae* is urn:lsid:zoobank.org:act:1AA0C22D-B579-4AF4-A904-75973A574EC3.

***Etymology*:** The new species is named after Patricia Mawson in recognition of her work on the taxonomy of macropodid nematodes.

### Description

**Fig. 3:**
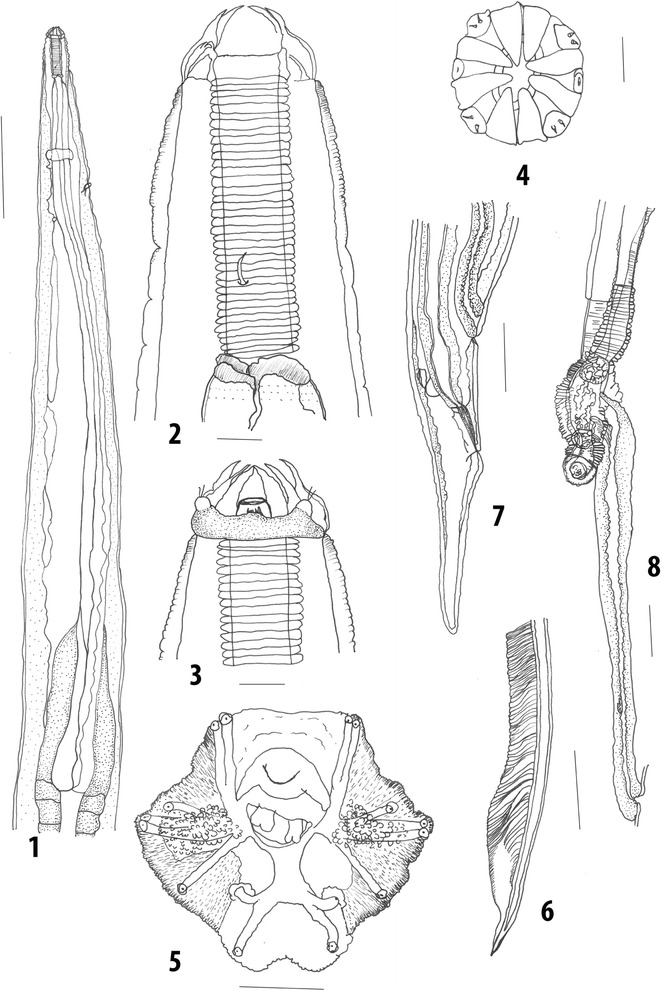
*Pharyngostrongylus patriciae* n. sp. from the stomach of *Osphranter robustus*. 1, Oesophagus, lateral view; 2, Buccal capsule, lateral view; 3, Buccal capsule and labial crown elements; 4, Oral opening apical view; 5, Bursa, apical view; 6, Spicule tip, ventral view; 7, Female tail, lateral view; 8, Vagina and ovejector, lateral view. *Scale-bars*: 1, 7, 8, 100 μm; 2–4, 10 μm; 5–6, 50 μm

***General*.** Small worm with numerous, very fine striations covering most of body, with fewer regularly spaced, broader transverse striations interspersed between them. No alae or longitudinal body striations. Cephalic collar demarcated posteriorly by transverse suture. Collar pierced by 2 amphids on conical projections and 4 conical, submedian papillae, each armed with 2 short setae. External labial crown of 8 petaloid elements; 2 arise between adjacent pairs of submedian papillae and 1 between each papilla and amphid. Labial crown elements continuous with external cephalic collar, with lining of buccal capsule internally. Mouth opening and buccal cavity circular in cross-section. Buccal capsule cylindrical, walls heavily sclerotized, of equal thickness, parallel, with broad, prominent, 38–50 regularly spaced transverse striations. Walls thinner at posterior extremity. Prominent extra chitinous supports present. Oesophagus long, narrow, lining smooth, bulb elongate, clavate. Intestinal wall extends anteriorly enveloping oesophageal bulb with paired, lateral prolongations. Deirids at level of buccal capsule. Nerve-ring encircles oesophagus near anterior end. Excretory pore posterior to nerve-ring (Fig. 3).

***Male*.** [Measurements of types; *n* = 10] Body length 5.55–7.86 (6.30), maximum width at mid-body 0.21–0.35 (0.29). Buccal capsule 0.08–0.10 (0.09) long, 0.02–0.03 (0.02) wide; oesophagus 1.44–1.74 (1.58) long. Distance from nerve-ring to anterior extremity 0.28–0.39 (0.32), from excretory pore to anterior extremity 0.34–0.45 (0.40), from deirid to anterior extremity 0.07–0.10 (0.09). Bursa short, lobes separated, dorsal lobe as long as lateral lobe with median indentation in margin. Surface of lateral lobes covered with numerous, dome-shaped, refractile bosses arranged in radial rows between striae. Ventral lobes covered with few small bosses. Spicules elongate, 2.25–1.98 (2.13) long, alate; alae with transverse striations, anterior extremities with irregular knob, posterior extremity blunt.

***Female*.** [Measurements of types; *n* = 10] Body length 7.24–10.25 (9.10), maximum width at mid-body 0.32–0.40 (0.39). Buccal capsule 0.09–0.12 (0.10) long, 0.02–0.03 (0.03) wide; oesophagus 1.05–2.08 (1.83) long. Distance from nerve-ring to anterior extremity 0.28–0.65 (0.38), from excretory pore to anterior extremity 0.32–0.92 (0.45), from deirid to anterior extremity 0.07–0.17 (0.10). Tail long, tapering gradually, 0.37–0.45 (0.39) long. Vulva immediately anterior to anus, distance to posterior extremity 0.65–0.75 (0.72); vagina elongate, 0.55–0.90 (0.74) long; ovejectors longitudinal, vaginae uterinae pass anteriorly from ovejectors. Eggs thin-shelled, ellipsoidal, none present in type-specimens.

### Remarks

The new species described here from *O. robustus* is attributed to *Pharyngostrongylus* based on its elongated and straight-walled buccal capsule with prominent transverse striations and a labial crown composed of petaloid elements. *Pharyngostrongylus patriciae* n. sp. can be differentiated from all congeners apart from *P. kappa* by the straight-sided, elongate buccal capsule with prominent transverse striations, eight labial crown elements and the lack of flap-like valves at the junction of the buccal capsule with the oesophagus. It is differentiated from *P. kappa* by the shorter buccal capsule, with 38–50 transverse striations instead of 60–64 striations in *P. kappa*.

### Molecular characterisation

Following sequencing, the flanking regions of the ITS sequences were trimmed based on sequences from GenBank [12] and the duplicate sequences were removed. A total of 28 and 29 unique sequences of ITS1 and ITS2 were identified, respectively. The trimmed sequences revealed that the ITS1 sequences of *P. kappa* (*n* = 15) and *P. patriciae* n. sp. (*n* = 14) ranged between 379–380 base pairs (bp) and 377–379 bp, respectively (Table 3). The ITS2 sequences of *P. kappa* (*n* = 17) ranged between 219–223 bp while those of *P. patriciae* n. sp. (*n* = 12) were 222 bp long (Table 3).

**Table 3:** Variation within sequences of internal transcribed spacers of *Pharyngostrongylus kappa* and *Pharyngostrongylus patriciae* from different hosts

Host	Voucher no.	ITS1				ITS2			
		GenBank ID	Length (bp)	G+C content (%)	Pairwise difference (%)	GenBank ID	Length (bp)	G+C content (%)	Pairwise difference (%)
*Pharyngostrongylus kappa*									
*M. giganteus*	1X8.1	MG972109	379	43.80	0.3–4.5	MG972110^a^	222	40.99	0.5–8.5
	1X8.2	MG972111^a^	379	45.90		MG972112	222	41.40	
	1X8.7	MG972113^b^	379	45.60		na	na	na	
	1X8.8	na	na	na		MG972114	222	40.54	
	7W1	na	na	na		MG972115^b^	219	42.00	
	7W2	MG972116	379	45.65		MG972117	223	40.36	
	P3A1	MG972118	380	45.79		MG972119^b^	219	42.00	
	P3A2	MG972120	379	46.17		na	na	na	
	P3A3	MG972121^a^	379	45.90		MG972122^c^	219	41.55	
	P5A1	MG972123	379	45.65		na	na	na	
	P7A2	na	na	na		MG972124	219	41.10	
	V3.1	na	na	na		MG972125	219	41.64	
	V3.2	MG972126^c^	379	45.91		MG972127^c^	219	41.55	
	V3.5	MG972128	379	45.65		na	na	na	
	V3.6	MG972129^d^	379	46.17		na	na	na	
	XV3.1	MG972130^d^	379	46.17		MG972131^c^	219	41.55	
	XV3.2	MG972132^b^	379	45.60		MG972133	222	40.99	
	XV3.4	MG972134	379	45.91		MG972135	219	41.10	
	XD1.4	MG972136^a^	379	45.90		MG972137	219	41.55	
	XD1.5	MG972138	379	45.38		MG972139	222	41.44	
	XD1.6	MG972140^c^	379	45.91		MG972141^c^	219	41.55	
	XD1.7	na	na	na		MG972142	219	41.10	
	WW3.5	na	na	na		MG927143^d^	222	41.00	
	WW3.6	MG972144^b^	379	45.60		na	na	na	
	XDR10.1	MG972145^c^	379	45.91		MG972146	219	41.10	
	XDR10.2	MG972147^b^	379	45.60		MG972148	222	41.44	
	XDR10.3	MG972149	379	45.38		MG972150^d^	222	41.00	
	XDR10.5	MG972151	379	46.44		na	na	na	
	XDR10.6	MG972152	379	45.65		MG972153^c^	219	41.55	
	XDR10.9	na	na	na		MG972154	219	42.00	
*Pharyngostrongylus patriciae* n. sp.									
*O. antilopinus*	27C3.2	MG972155^e^	378	45.50	0.6	na	na	na	na
	28A1	MG972156	378	45.50		MG972157^e^	222	42.34	
	38T.31	na	na	na		MG972158^e^	222	42.34	
*O. robustus*	21J3.1	MG972159	378	45.24	0.3–3.2	MG972160	222	42.34	1.0–5.0
	21J3.2	MG972161	378	44.71		MG972162	222	41.44	
	21J3.5	MG972163	378	45.77		MG972164	222	40.99	
	21J3.6	na	na	na		MG972165	222	40.99	
	21J3.13	na	na	na		MG972166	222	42.34	
	21J3.14	MG972167	378	45.77		MG972168^a^	222	40.99	
	26Z1.10	MG972169^f^	378	44.97		na	na	N/A	
	27G9.6	MG972170	378	44.97		MG972171	222	41.89	
	27G9.7	MG972172	378	45.24		MG972173^e^	222	42.34	
	27G9.8	MG972174	377	44.83		MG972175	222	40.54	
	27G9.11	MG972176^e^	378	45.50		na	na	42.30	
	38V15	MG972177^f^	378	44.97		MG972178	222	42.34	
	38V18	MG972179^e^	378	45.50		MG972180^e^	222	42.34	
*O. bernardus*	28C2	na	na	na	0.3–2.7	MG972181	222	41.44	0.5–2.3
	28C3	MG972182	378	44.44		MG972183	222	41.44	
	28C4	na	na	na		MG972184	222	41.89	
	28C17	MG972185	379	45.91		na	na	na	
	28C20	MG972186	379	45.91		na	na	na	
	28C21	MG972187^f^	378	44.97		na	na	na	

*Abbreviations*: *G* guanine, *C* cytosine, *ITS1* first internal transcribed spacer, *ITS2* second internal transcribed spacer, *na* unable to determine due to unsuccessful DNA amplification or poor-quality sequences

^a-f^The same superscripts for GenBank IDs indicate identical sequences

The ITS1 and ITS2 sequences of both *P. kappa* and *P. patriciae* n. sp. determined herein were aligned (separately) with those previously published by Chilton et al. [12] over 384 and 227 positions, respectively. Specimen XDR10.1 (GenBank: MG972145, MG972146) from *M. giganteus*, Inglewood, Queensland was chosen as the reference sequence as it is from the type-host and locality of *P. kappa* (Additional file 1: Figures S2 and S3). The ITS1 sequences from *M. giganteus* had fewer variations than those from *Osphranter* spp. Contrarily, the ITS2 sequences from *Osphranter* spp. exhibited greater variation compared to those from *M. giganteus*. However, there were consistent differences in nucleotide bases between individuals from *M. giganteus* and those from *Osphranter* spp. in both ITS1 and ITS2 regions. The only exceptions were the ITS2 sequences MG972110 and MG972112 from *M. giganteus* at Clermont, Queensland which shared the same sequences as specimens from *Osphranter* spp.

Overall pairwise nucleotide differences in the ITS2 sequences were higher (0.5–8.5%) than those of ITS1 (0.3–4.5%) (Table 3). The greater difference was attributed to the high similarity (97–99%) between ITS2 sequences of specimens MG972110 and MG972112 from *M. giganteus*, from Clermont, Queensland to those of specimens from *O. robustus* in Charters Towers, Queensland (see Additional file 1: Figures S2 and S3).

### Phylogenetic analysis

**Fig. 4:**
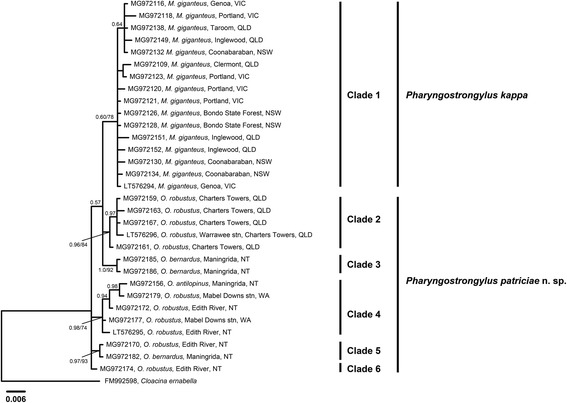
Phylogenetic analysis of the ITS1 rDNA sequences of *Pharyngostrongylus kappa* and *Pharyngostrongylus patriciae* n. sp. from various host species and geographical locations. The sequence data were analysed using the Neighbour-Joining (NJ) and Bayesian Inference (BI) methods. There was a concordance between the topology of the BI tree and the NJ tree (not shown). Nodal support is given as a posterior probability of BI/bootstrap value for NJ. Each unique sequence is presented with a GenBank accession no. followed by the voucher number, and its host and locality. Three sequences (LT576294-LT576296) were included as reference sequences from Chilton et al. [12]. *Cloacina ernabella* was used as the outgroup. Scale-bar indicates the number of inferred substitutions per nucleotide site. *Abbreviations*: NSW, New South Wales; NT, Northern Territory; QLD, Queensland; stn, station; VIC, Victoria; WA, Western Australia

**Fig. 5:**
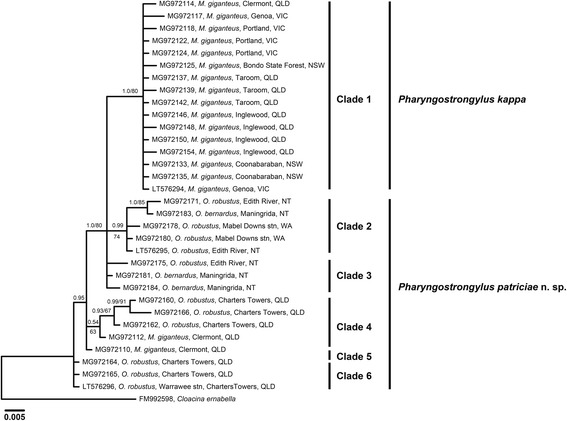
Phylogenetic analysis of the ITS2 rDNA sequences of *Pharyngostrongylus kappa* and *Pharyngostrongylus patriciae* n. sp. from various host species and geographical locations. The sequence data were analysed using the Neighbour-Joining (NJ) and Bayesian Inference (BI) methods. There was a concordance between the topology of the BI tree and the NJ tree (not shown). Nodal support is given as a posterior probability of BI/bootstrap value for NJ. Each unique sequence is presented with a GenBank accession no. followed by the voucher number, and its host and locality. Three sequences (LT576294-LT576296) were included as reference sequences from Chilton et al. [12]. *Cloacina ernabella* was used as the outgroup. Scale-bar indicates the number of inferred substitutions per nucleotide site. *Abbreviations*: NSW, New South Wales; NT, Northern Territory; QLD, Queensland; stn, station; VIC, Victoria; WA, Western Australia

**Fig. 6:**
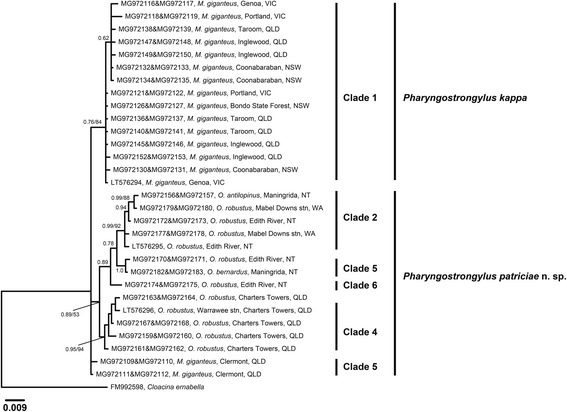
Phylogenetic analysis of the ITS+ rDNA sequences of *Pharyngostrongylus kappa* and *Pharyngostrongylus patriciae* n. sp. from various host species and geographical locations. The sequence data were analysed using the Neighbour-Joining (NJ) and Bayesian Inference (BI) methods. There was a concordance between the topology of the BI tree and the NJ tree (not shown). Nodal support is given as a posterior probability of BI/bootstrap value for NJ. Unique sequences are presented with a GenBank accession no. followed by the voucher number, and host and locality. Three sequences (LT576294-LT576296) were included as reference sequences from Chilton et al. [12]. *Cloacina ernabella* was used as the outgroup. Scale-bar indicates the number of inferred substitutions per nucleotide site. *Abbreviations*: NSW, New South Wales; NT, Northern Territory; QLD, Queensland; stn, station; VIC, Victoria; WA, Western Australia

**Fig. 7:**
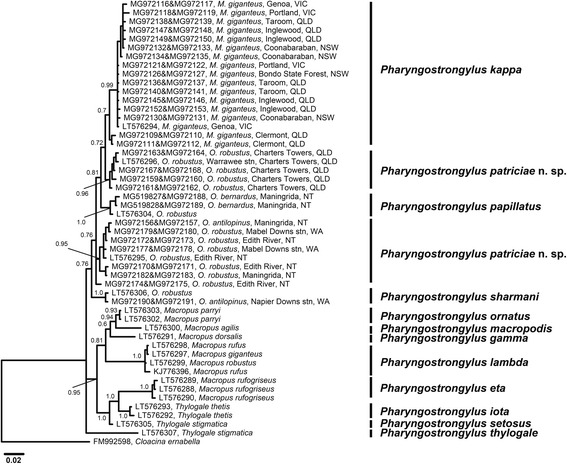
Phylogenetic analysis of the concatenated ITS+ rDNA sequences of *Pharyngostrongylus* spp. from various host species and geographical locations. The sequence data were analysed using the Neighbour-Joining (NJ) and Bayesian Inference (BI) methods. There was a concordance between the topology of the BI tree and the NJ tree (not shown). Nodal support is given as a posterior probability of BI. *Cloacina ernabella* was used as the outgroup. Scale-bar indicates the number of inferred substitutions per nucleotide site. *Abbreviations*: NSW, New South Wales; NT, Northern Territory; QLD, Queensland; VIC, Victoria; WA, Western Australia

The ITS1, ITS2 and concatenated ITS (ITS+) sequence data of *P. kappa* and *P. patriciae* n. sp. were analysed using the Neighbour-Joining (NJ) and Bayesian Inference (BI) methods. All the NJ and BI trees produced were similar in topology, hence only BI trees are presented for ITS1 (Fig. 4), ITS2 (Fig. 5) and ITS+ (Fig. 6) from *P. kappa* and *P. patriciae* datasets. Phylogenetic trees of ITS+ sequences of *P. kappa* and *P. patriciae* with *P. papillatus*, *P. sharmani* and all available congener sequences were produced using both BI and NJ methods (Fig. 7).

Phylogenetic analysis of the ITS1 sequences (Fig. 4) displayed six major clades. Clade 1 contained all sequences of *P. kappa* from *M. giganteus* with weak nodal support (posterior probabilities for BI = 0.60; bootstrap value for NJ = 78%). Within this clade, a sub-clade contained sequences from NSW, Qld and Vic, with weak nodal support (BI, 0.64). Clade 2 contained all sequences from *O. robustus* at Charters Towers, Qld, with moderate nodal support (BI, 0.96; NJ, 84%) and one sequence (MG972161) diverging from the main clade. Two sequences from *O. bernardus* from Maningrida, NT, formed Clade 3, with strong statistical support (BI, 1.0; NJ, 92%). Clade 4 contained sequences from all three species of *Osphranter* from NT and sequences from *O. robustus* and *O. antilopinus* in WA, with moderate support (BI, 0.98; NJ, 74%). Sequences from *O. robustus* at Edith River and *O. bernardus* from Maningrida, NT formed a fifth clade with strong statistical support (BI, 0.97; NJ, 93%). One sequence of *O. robustus* from Edith River, diverged in its own clade. Overall, clade formation was correlated with host species rather than the collection localities. Based on the number of clades, specimens of *P. patriciae* n. sp. exhibited greater genetic diversity than those of *P. kappa*.

Phylogenetic analysis of the ITS2 sequences yielded a tree with similar topology to ITS1 sequences with six major clades (Fig. 5). The major difference in the topology of the ITS2 tree were two sequences from *M. giganteus* in Clermont, Qld (MG972110 and MG972112) that grouped outside the *M. giganteus* clade. Instead, these two sequences clustered with the *O. robustus* clade from Charters Towers, Qld, though with weak nodal support (BI, 0.54; NJ, 63%).

Phylogenetic analysis of concatenated ITS sequences displayed a tree (Fig. 6) that shared a similar topology to both ITS1 and ITS2 trees. The ITS+ sequences from *M. giganteus* formed an identical clade and sub-clade to Clade 1 in the ITS1 tree. As with the ITS2 tree, the *M. giganteus* sequences from Clermont, Qld diverged forming an isolated clade (Fig. 6). Among the ITS+ sequences from *Osphranter* spp., *O. robustus* sequences from Charters Towers, Qld formed a separate clade (Clade 4) to the remaining sequences from the NT and WA (Fig. 6).

Phylogenetic analysis of concatenated ITS sequences of *P. papillatus* (GenBank: MG519827-MG519828 and MG972188-MG972189) and *P. sharmani* (GenBank: MG972190-MG972191) determined here, and all of those available on GenBank sequences of congeners [12, 30] produced a tree (Fig. 7) with a similar topology to the ITS+ analyses of *P. kappa* and *P. patriciae* n. sp*.* The ITS+ sequences of *P. kappa* formed one clade (nodal support BI, 0.99 and 0.7) while those of *P. patriciae* diverged into two clades (nodal support BI, 0.96 and 0.95). The ITS+ sequences of *P. papillatus* formed one clade with strong statistical support (BI, 1.0) in between two clades of *P. patriciae.* The ITS+ sequences of *P. sharmani* also formed a clade with strong statistical support (BI, 1.0). Each *Pharyngostrongylus* sp. from GenBank formed a separate clade with strong statistical support (see Fig. 7).

Based on phylogenetic analyses of ITS sequences of all *Pharyngostrongylus* spp., genetic variation was associated with differences in hosts rather than with geographical localities, with one exception, the two sequences of *P. kappa* from *M. giganteus* from Clermont that formed an isolated sub-clade within the *P. kappa* clade (see Fig. 7).

## Discussion

The present study aimed to characterise the genetic and morphological diversity of *P. kappa* from its macropodid hosts, including *Macropus giganteus*, *Osphranter robustus*, *O. antilopinus* and *O. bernardus*. It was hypothesised that due to the wide host range and geographical distribution, there was potential for intraspecific genetic variation within *P. kappa*. Molecular and morphological findings provide evidence that specimens of *P. kappa* from different host genera are genetically and morphologically distinct from one another.

*Pharyngostrongylus kappa* from *Osphranter* spp. are now recognised as a new species based on the length of the buccal capsule and the number of transverse striations. Specimens from *Osphranter* spp. had shorter buccal capsules with fewer striations compared to specimens from *M. giganteus*. Measurements show that the buccal capsule length of *P. kappa* from *M. giganteus* (0.12–0.17 mm) fell within range of the type-specimens (0.13–0.19 mm) described by Mawson [31]. However, the buccal capsule length of specimens from *Osphranter* spp. fell below this range [*O. robustus* (type-specimens): 0.08–0.10 mm; *O. antilopinus*: 0.08–0.12 mm; and *O. bernardus*: 0.09–0.11 mm]. The present study also found consistent differences in the number of striations on the buccal capsule of *P. kappa* from *M. giganteus* (60–40 striations) and *P. patriciae* n. sp. from *Osphranter* spp. (38–50 striations). Although this feature was not recorded in the type-specimens nor the redescription by Beveridge [11], it could be considered taxonomically significant for other members of this genus. However, the morphological differences observed in specimens of *P. patriciae* n. sp. from *Osphranter* spp. are supported by molecular evidence strengthening the hypothesis that specimens from *Osphranter* spp. represent a new species of *Pharyngostrongylus*.

Sequence datasets of the ITS rDNA indicate that *P. kappa* occurring in the primary host, *M. giganteus* is genetically distinct from those occurring in *O. robustus*, *O. antilopinus* and *O. bernardus.* The length of the ITS2 sequence of *P. kappa* from *M. giganteus* from Genoa, Victoria (219 bp) is identical to the published sequence (GenBank: LT576294; [12]) from the same host and location. The ITS1 sequence amplification was unsuccessful, hence there was no comparable data for this region. Both the ITS1 and ITS2 of *P. patriciae* specimens from *O. robustus* from Charters Towers in Queensland were identical to a published sequence (GenBank: LT576296; [12]). In contrast, the lengths of ITS1 (387 bp) and ITS2 (222 bp) of *P. patriciae* from *O. robustus* from Edith River in the Northern Territory differed by one base pair from the published sequence (GenBank: LT576295; [12]) from the same host and locality (ITS1, 386 bp; ITS2, 223 bp). These differences could be attributed to the larger sample size included in the current study.

Phylogenetic analysis of the ITS sequences indicated that specimens of *P. kappa* from *M. giganteus* and those of *P. patriciae* from *Osphranter* spp. did not form a monophyletic assemblage, providing support for earlier work by Chilton et al. [12]. Furthermore, ITS+ sequences of *P. patriciae* from *O. robustus* from Charters Towers, Queensland, formed a separate clade from specimens in *Osphranter* spp. from Western Australia and the Northern Territory also consistent with the study of Chilton et al. [12].

The populations of nematodes studied were taken from two different subspecies of *M. robustus*: *M. r. robustus* from the Charters Towers region of Queensland, and *M. r. woodwardi* from the Northern Territory and Western Australia [1] and may represent co-divergence with the two host subspecies. In addition, the two nematode populations appear to be disjunct as *P. patriciae* was not found in six *M. robustus* examined in the Mount Isa-Cloncurry region of north-western Queensland (I. Beveridge, unpublished data).

The only exception to the general pattern of clade formation was the segregation of two ITS+ sequences of *P. kappa* from *M. giganteus* from Clermont, Queensland (MG972110 and MG972112). The sharing of nucleotide changes in the ITS1 with specimens from *M. giganteus* and ITS2 with specimens of *P. patriciae* n. sp. from *O. robustus* may be indicative of genetic introgression. Clermont is in an area where *M. giganteus* occurs in sympatry with *O. robustus*. *Pharyngostrongylus kappa* may have switched hosts, resulting in the combination of ITS1 and ITS2 nucleotide changes observed. All specimens of *P. kappa* from Clermont were morphologically identical and conformed to the phenotype normally found in *M. giganteus*. Chilton et al. [15] found evidence of hybridisation between *Paramacropostrongylus iugalis* occurring in *M. giganteus* and *P. typicus* in *M. fuliginosus* in an area of host sympatry. However, there is insufficient evidence in this study to support the hypothesis of hybridisation among genetically distinct forms of *P. kappa*. Evidence from previous studies suggests that speciation of nematodes in macropodid marsupial hosts has occurred primarily by host-switching [7]. However, a larger sample size of *P. kappa* and *P. patriciae* from hosts within areas of sympatry with *Osphranter* spp. is required to confirm the occurrence of such host-switching events.

The current study also sequenced congeners of *P. kappa*, *P. papillatus* and *P. sharmani* from northern macropodid species. The occurrence of *P. papillatus* in *O. bernardus* documented in this study represents a new host record as *P. papillatus* has previously been found in *O. antilopinus* and *O. robustus* [18]. Additionally, ITS+ sequences of *P. papillatus* from *O. bernardus* formed distinct clades from specimens from *O. robustus*. These results suggest a potential cryptic species occurring among *P. papillatus* that require further morphological and molecular analysis.

## Conclusions

The evidence from the current study supports the hypothesis that *P. kappa* found in *M. giganteus* is genetically distinct from *P. patriciae* n. sp. found in *Osphranter* spp. Furthermore, morphological data revealed distinctive features of specimens from *Osphranter* spp. supporting the morphological data. The description of a new species was made based on morphological and molecular evidence. This study contributes to earlier efforts in documenting the diversity of Australian nematodes and suggests that molecular techniques indicate that the diversity requires further detailed exploration.

## Additional file

Additional file 1: **Table S1.** Specimens of *Pharyngostrongylus kappa* from *Macropus giganteus* examined from the South Australian Museum (SAM), Adelaide. **Figure S1.** Alignment of ITS1 rDNA sequences of *Pharyngostrongylus kappa* and *P. patriciae* n. sp. from different macropodid hosts. A dot indicates an identical nucleotide with respect to the sequence of XDR10.1; a dash indicates an insertion/deletion (indel) event. IUPAC codes indicate polymorphic positions in the sequences. **Figure S2.** Alignment of ITS2 rDNA sequences of *Pharyngostrongylus kappa* and *P. patriciae* n. sp. from different macropodid hosts. A dot indicates an identical nucleotide with respect to the sequence of XDR10.1; a dash indicates an insertion/deletion (indel) event. IUPAC codes indicate polymorphic positions in the sequences. (DOCX 69 kb)
