# Supplementary material for: Molecular and morphological characterisation of Pharyngostrongylus kappa Mawson, 1965 (Nematoda: Strongylida) from Australian macropodid marsupials with the description of a new species, P. patriciae n. sp
Source: Parasit Vectors. 2018 Apr 27;11:271. doi: 10.1186/s13071-018-2816-6 (PMC5924500; doi:10.1186/s13071-018-2816-6)
Supplement: Supplementary file 1 — Table S1. Specimens of Pharyngostrongylus kappa from Macropus giganteus examined from the South Australian Museum (SAM), Adelaide. Figure S1. Alignment of ITS1 rDNA sequences of Pharyngostrongylus kappa and P. patriciae n. sp. from different macropodid hosts. A dot indicates an identical nucleotide with respect to the sequence of XDR10.1; a dash indicates an insertion/deletion (indel) event. IUPAC codes indicate polymorphic positions in the sequences. Figure S2. Alignment of ITS2 rDNA sequences of Pharyngostrongylus kappa and P. patriciae n. sp. from different macropodid hosts. A dot indicates an identical nucleotide with respect to the sequence of XDR10.1; a dash indicates an insertion/deletion (indel) event. IUPAC codes indicate polymorphic positions in the sequences. (DOCX 69 kb) [file 13071_2018_2816_MOESM1_ESM.docx]

**Additional file 1. Table S1.** Specimens of *Pharyngostrongylus kappa* from *Macropus giganteus* examined from the South Australian Museum (SAM), Adelaide

| SAM  reg. | Hosts | Localities | Coordinates | No. of males | No. of females |
| --- | --- | --- | --- | --- | --- |
| 7279 | *M. giganteus* | Townsville, Qld | 19°15′S, 146°49′E | 3 | 5 |
| 7353 | *M. giganteus* | Woodstock, Qld | 19°39′S, 146°51′E | 17 | 10 |
| 7397 | *M. giganteus* | Charters Towers, Qld | 20°4′S, 146°15′E | 5 | 4 |
| 7677 | *M. giganteus* | Harvey’s Range, Townsville, Qld | 19°20′S, 146°29′E | 12 | 18 |
| 8649 | *M. giganteus* | Armidale, NSW | 30°30′S, 151°29′E | 5 | 12 |
| 9215 | *M. giganteus* | Dartmouth, Vic | 36°31′S, 147°29′E | 8 | 17 |
| 9275 | *M. giganteus* | Zumsteins, Vic | 37°37′S, 142°23′E | 6 | 3 |
| 9620 | *M. giganteus* | Yan Yean, Vic | 37°34′S, 145°6′E | 13 | 8 |
| 9709 | *M. giganteus* | Marlo Plains, Vic | 37°46′S, 148°40′E | 14 | 13 |
| 10528 | *M. giganteus* | Kingstown, NSW | 30°31′S, 151°7′E | 0 | 1 |
| 10599 | *M. giganteus* | Pilliga, NSW | 30°21′S, 148°53′E | 2 | 1 |
| 10606 | *M. giganteus* | Kingstown, NSW | 30°31′S, 151°7′E | 15 | 4 |
| 10904 | *M. giganteus* | Mirranatwa, Vic | 37°24′S, 142°24′E | 1 | 1 |
| 10939 | *M. giganteus* | Tidbinbilla, ACT | 35°26′S, 148°56′E | 12 | 14 |
| 11047 | *M. giganteus* | Fraser National Park, Vic | 37°19′S, 146°0′E | 0 | 1 |
| 11062 | *M. giganteus* | Rockhampton, Qld | 23°22′S, 150°30′E | 15 | 13 |
| 12300 | *M. giganteus* | Warrawee Stn, Charters Towers, Qld | 20°4′S, 146°15′E | 0 | 1 |
| 13380 | *M. giganteus* | Harvest Home Stn Charters Towers, Qld | 20°4′S, 146°15′E | 0 | 1 |
| 13495 | *M. giganteus* | Harvest Home Stn Charters Towers, Qld | 20°4′S, 146°15′E | 6 | 11 |
| 13496 | *M. giganteus* | Harvest Home Stn Charters Towers, Qld | 20°4′S, 146°15′E | 0 | 3 |
| 19899 | *M. giganteus* | Bondo State Forest, NSW | 35°17′S, 148°34′E | 22 | 13 |
| 19901 | *M. giganteus* | Melmoth Stn Dingo, Qld | 23°38′S, 149°20′E | 2 | 7 |
| 19902 | *M. giganteus* | Banana, Qld | 24°28′S, 150°7′E | 1 | 5 |
| 19907 | *M. giganteus* | Killarney, Qld | 28°19′S, 152°17′E | 8 | 8 |
| 23223 | *M. giganteus* | 50 km E of Inglewood, Qld | 28°24′S, 151°4′E | 14 | 13 |
| 23230 | *M. giganteus* | 12 km S of Theodore, Qld | 24°56′S, 150°4′E | 2 | 2 |
| 23234 | *M. giganteus* | 10 km west of Mungallala, Qld | 26°26′S, 147°32′E | 21 | 11 |
| 23241 | *M. giganteus* | 70 km N of Coonabarabran, NSW | 31°16′S, 149°13′E | 19 | 10 |
| 24246 | *M. giganteus* | 40 km N of Charters Towers, Qld | 20°4′S, 146°15′E | 1 | 0 |
| 24254 | *M. giganteus* | Bogantungan, Qld | 23°38′S, 147°17′E | 6 | 8 |
| 24275 | *M. giganteus* | 35 km S of Clermont, Qld | 22°49′S, 147°37′E | 0 | 1 |
| 24691 | *M. giganteus* | Gilgandra, NSW | 31°41′S, 148°39′E | 0 | 2 |
| 25698 | *M. giganteus* | 66 km E of Moonie, Qld | 27°43′S, 150°22′E | 2 | 2 |
| 31551 | *M. giganteus* | Portland, Vic | 38°21′S, 141°36′E | 7 | 4 |
| 34620 | *M. giganteus* | Lara, Vic | 38°1′S, 144°25′E | 2 | 3 |

**Additional file 1. Figure S1**

10 20 30 40 50 60 70 80 90 100

....|....|....|....|....|....|....|....|....|....|....|....|....|....|....|....|....|....|....|....|

**XDR10.1**  **TCGAAACCTTATGGTTCCCTTGATCTTGAGAAACCAACATGCTTGATACTTCACGACTTTGTTCGTGAAGAGTTGGGAGTATCAGCCCAC--CTTTTTAA**

**1X8.1**  **.............................S....Y.......................................................--........**

**7W2**  **..........................................................................................--........**

**P3A1**  **..........................................................................................--........**

**P3A2**  **..........................................................................................--........**

**P3A3**  **..........................................................................................--........**

**P5A1**  **..........................................................................................--........**

**V3.5**  **..........................................................................................--........**

**XD1.5**  **..........................................................................................--........**

**XDR10.3**  **..........................................................................................--........**

**XDR10.5**  **..........................................................................................--........**

**XDR10.6**  **..........................................................................................--........**

**XV3.1**  **..........................................................................................--........**

**XV3.2**  **..........................................................................................--........**

**XV3.4**  **..........................................................................................--........**

**LT576294** **..........................................................................................--........**

**21J3.1**  **..................T.......................................................................--........**

**21J3.2**  **..................T.................................................................S.....--........**

**21J3.5**  **..................T.......................................................................--........**

**21J3.14**  **..................T.......................................................................--........**

**27G9.6**  **..................T.......................................................................--........**

**27G9.7**  **..................T.......................................................................--........**

**27G9.8**  **.SR...............T.......................................................................--........**

**38V15**  **..................T.......................................................................--........**

**38V18**  **..................T.......................................................................--........**

**28A1**  **..................T.......................................................................--........**

**27C3.2**  **..................T.......................................................................--........**

**28C3**  **..................T.....Y.................................................................--........**

**28C17**  **..................T....................................G..................................--........**

**28C20**  **..................T....................................G..................................--........**

**LT576295** **..................T......................................................................TCG........**

**LT576296** **..................TY......................................................................--........**

110 120 130 140 150 160 170 180 190 200

....|....|....|....|....|....|....|....|....|....|....|....|....|....|....|....|....|....|....|....|

**XDR10.1**  **TCCGTTTGGAGGTGTCTATGTACAACATGAGCCGTTTGCGGATGGCGTCAGTGATTGTTGTGCAAAGTTCGCGCATTCAGTTGTGCTGAGCTWTAGACTT**

**1X8.1**  **..................................................A.............................................MMY.**

**7W2**  **....................................................................................................**

**P3A1**  **....................................................................................................**

**P3A2**  **....................................................................................................**

**P3A3**  **....................................................................................................**

**P5A1**  **....................................................................................................**

**V3.5**  **..................................................R.................................................**

**XD1.5**  **...............................................................................K....................**

**XDR10.3**  **....................................................................................................**

**XDR10.5**  **............................................................................................GC......**

**XDR10.6**  **.T..................................................................................................**

**XV3.1**  **..................G.................................................................................**

**XV3.2**  **....................................................................................................**

**XV3.4**  **....................................................................................................**

**LT576294** **....................................................................................................**

**21J3.1**  **..................A..S.........................................................S....................**

**21J3.2**  **..................A.................................................................................**

**21J3.5**  **..................A..G..............................................................................**

**21J3.14**  **..................A..G..............................................................................**

**27G9.6**  **..................A..............................T..................................................**

**27G9.7**  **..................A..............................T...........................................C......**

**27G9.8**  **..................A..............................T.............................A-...................**

**38V15**  **..................A..............................T..................................................**

**38V18**  **..................A..............................T...........................................C......**

**28A1**  **..................A..............................T...........................................C......**

**27C3.2**  **..................A..............................T...........................................C......**

**28C3**  **...............Y..A..............................T..................................................**

**28C17**  **..................W............................................G....................................**

**28C20**  **..................A............................................G....................................**

**LT576295** **..................A..............................T..................................................**

**LT576296** **..................A..G.........................................................C....................**

210 220 230 240 250 260 270 280 290 300

....|....|....|....|....|....|....|....|....|....|....|....|....|....|....|....|....|....|....|....|

**XDR10.1**  **GATGAGCACTGCATGAGTGCCGCCTCTATGTTTGTACTGGTGGTTAGGCATTAGGCTGTCACCAGCCTTCTGCAACACCTGCTGGTCAGGAAATCTCAAT**

**1X8.1**  **S.....MR.......................K.......................S............................................**

**7W2**  **....................................................................................................**

**P3A1**  **............................................................G.......................................**

**P3A2**  **....................................................................................................**

**P3A3**  **....................................................................................................**

**P5A1**  **....................................................................................................**

**V3.5**  **....................................................................................................**

**XD1.5**  **....................................................................................................**

**XDR10.3**  **....................................................................................................**

**XDR10.5**  **....................................................................................................**

**XDR10.6**  **....................................................................................................**

**XV3.1**  **....................................................................................................**

**XV3.2**  **....................................................................................................**

**XV3.4**  **....................................................................................................**

**LT576294** **....................................................................................................**

**21J3.1**  **.........................................................C......................................T...**

**21J3.2**  **.......R.............................................K...C......................................T...**

**21J3.5**  **.........................................................C......................................T...**

**21J3.14**  **.........................................................C......................................T...**

**27G9.6**  **........T.......................................................................................T...**

**27G9.7**  **.........................................................C.A....................................T...**

**27G9.8**  **................................................................................................T...**

**38V15**  **.........................................................C.A....................................T...**

**38V18**  **.........................................................C.A....................................T...**

**28A1**  **..................................................C......C.A....................................T...**

**27C3.2**  **.........................................................C.A....................................T...**

**28C3**  **........T.......................................................................................T...**

**28C17**  **................................................................................................T...**

**28C20**  **................................................................................................T...**

**LT576295** **.........................................................S.A....................................T...**

**LT576296** **.........................................................C......................................T...**

310 320 330 340 350 360 370 380

....|....|....|....|....|....|....|....|....|....|....|....|....|....|....|....|....

**XDR10.1**  **GACTCGTACACCGAGCGCCAGTAC-AGATGACAACATTTTACATTTGA-CGTTTGAAGAATCGTGACTTTA-TGTCACAATCGA**

**1X8.1**  **......W.W...............-.......................-......................-............**

**7W2**  **........W...............-.......................-......................-............**

**P3A1**  **........T...............TT......................-......................-............**

**P3A2**  **........................-..............C........-......................-............**

**P3A3**  **........................-.......................-......................-............**

**P5A1**  **........T...............-.......................-......................-............**

**V3.5**  **........................-.......................-......................-............**

**XD1.5**  **........T...............-.......................-......................-............**

**XDR10.3**  **........T...............-.......................-........A.............-............**

**XDR10.5**  **........................-.......................-......................-............**

**XDR10.6**  **........................-.......................-......................-............**

**XV3.1**  **........................-.......................-......................-............**

**XV3.2**  **........T...............-.......................-......................-............**

**XV3.4**  **........................-.......................-......................-............**

**LT576294** **........................-.......................-......................-............**

**21J3.1**  **........................-.....-.................-......................-............**

**21J3.2**  **..............S.........-.....-.................-......................-............**

**21J3.5**  **........................-.....-.................-.....................T-............**

**21J3.14**  **........................-.....-.................-......................-............**

**27G9.6**  **........................-....-..................-......................-............**

**27G9.7**  **.......T................-....-.........Y........-......................-............**

**27G9.8**  **.......T................-.....-.................-......................-............**

**38V15**  **.......T................-....-..................-......................-............**

**38V18**  **.......T................-....-..................-...................C..-............**

**28A1**  **.......T................-....-..................-...................Y..-............**

**27C3.2**  **.......T................-....-..................-...................C..-............**

**28C3**  **........................-....-..................-......................-............**

**28C17**  **.......T................-.......................-......................-............**

**28C20**  **.......T................-.......................-......................-............**

**LT576295** **.......T................-.......................C......................T............**

**LT576296** **........................-.....-.................-......................-............**

**Figure S1** Alignment of the ITS-1 rDNA sequences of *Pharyngostrongylus kappa* and *Pharyngostrongylus patriciae* from different macropodid hosts. A dot indicates an identical nucleotide with respect to the sequence of XDR10.1; a dash indicates an insertion/deletion (indel) event. IUPAC codes indicate polymorphic positions in the sequences.

**Additional file 1: Figure S2**

10 20 30 40 50 60 70 80 90 100

....|....|....|....|....|....|....|....|....|....|....|....|....|....|....|....|....|....|....|....|

**XDR10.1**  **TTCAATATACTACAGTGTGGCCTG-TATAACACTGTTTGTCGAATGACGCTTGTGCAT--TATRTGCGCAATCCTCGTTCTAGATGAGAACTATATTGCA**

**1X8.1**  **........................-.................................TT........................................**

**1X8.2**  **........................-.................................TT...............................Y........**

**1X8.8**  **........................-.................................--......................................T.**

**7W2**  **.........A.....M........N.................................--Y.......................................**

**P3A1**  **........................-.................................--........................................**

**P3A3**  **........................-.................................--........................................**

**P7A2**  **........................-.................................--................R.......................**

**V3.1**  **...........R.....C...Y..-..Y....Y.........................--........................................**

**XD1.4**  **........................-.................................--........................................**

**XD1.5**  **........................-.................................--...............................C........**

**XD1.7**  **........................-.................................--........................................**

**XDR10.2**  **........................-.................................--................................G.......**

**XDR10.3**  **........................-.................................--........................................**

**XDR10.9**  **........................-.................................--........................................**

**XV3.2**  **........................-.................................--........................................**

**XV3.4**  **........................-.................................--........................................**

**LT576294**  **........................-.................................--........................................**

**21J3.1**  **........................-.................................TC...............................C........**

**21J3.2**  **........................-.................................TT...............................C........**

**21J3.5**  **........................-.................................TT...............................Y........**

**21J3.6**  **........................-.................................TT........................................**

**21J3.13**  **........................-.................................TC...............................C........**

**27G9.6**  **........................-.................................GTC.....T.................................**

**27G9.8**  **........................-.................................TT........................................**

**38V.15**  **........................-.................................GT..........R.............................**

**38V.18**  **........................-.................................GT........................................**

**28C2**  **........................-.................................TT........................................**

**28C3**  **........................-.................................GT......T.................................**

**28C4**  **........................-.................................TT.......................G................**

**LT576295**  **........................-.................................GT........................................**

**LT576296**  **........................-.................................TT........................................**

110 120 130 140 150 160 170 180 190 200

....|....|....|....|....|....|....|....|....|....|....|....|....|....|....|....|....|....|....|....|

**XDR10.1**  **ACATGTAC---CATTATGTGGTACGCGATTGTCATGACCGTGTTGTCACTGTCAAAAGTATTTAGCGAGAAATGAATACTTTGGCGGGGCTTGTTTGWGA**

**1X8.1**  **.......T---...C.........A...A..........................................T.......................A....**

**1X8.2**  **.......T---...C.........A...A..........................................T.......................A....**

**1X8.8**  **........CAT.........................................................................................**

**7W2**  **........CAT.........................................................................................**

**P3A1**  **........---.........................................................................................**

**P3A3**  **........---.........................................................................................**

**P7A2**  **........---.........................................................................................**

**V3.1**  **........---.........................................................................................**

**XD1.4**  **........---.........................................................................................**

**XD1.5**  **........CAT.........................................................................................**

**XD1.7**  **........---......K..................................................................................**

**XDR10.2**  **........CAT.........................................................................................**

**XDR10.3**  **........CAT.........................................................................................**

**XDR10.9**  **........---.........................................................................................**

**XV3.2**  **........CAT.............................................................K...........................**

**XV3.4**  **........---.........................................................................................**

**LT576294**  **........---.........................................................................................**

**21J3.1**  **.......T---...C.........A...A..........................................T.......................A....**

**21J3.2**  **.......T---...C.........A...A..........................................T.......................A....**

**21J3.5**  **.......T---...C.........A...A.........................................TT.......................A....**

**21J3.6**  **.......T---...C.........A...A.........................................TT.......................A....**

**21J3.13**  **.......T---...C.........A...A.T...C....................................T.......................A....**

**27G9.6**  **.......T---...C.........A...A.............................................................C....A....**

**27G9.8**  **........---...C........TA...A.......................................T..........................A....**

**38V.15**  **........---...C.S.......A...A.............................................................C....A....**

**38V.18**  **........---...C.........A...A.............................................................C....A....**

**28C2**  **........---...C.........A...A..................................................................A....**

**28C3**  **.......T---...C.........A...A.............................................................C....A....**

**28C4**  **........---...C.........A...A..................................................................A....**

**LT576295**  **........---...C.........A...A.............................................................C....A....**

**LT576296**  **.......T---...C.........A...A.........................................TT.......................A....**

210 220

....|....|....|....|....|..

**XDR10.1**  **CAACGACG-GTC-TATGTCATTTGCAA**

**1X8.1**  **........C...-..............**

**1X8.2**  **........C...-.R............**

**1X8.8**  **........-...-..............**

**7W2**  **........-...-..............**

**P3A1**  **.G......-...-..............**

**P3A3**  **........-...-..............**

**P7A2**  **........-...-..............**

**V3.1**  **........-...-..............**

**XD1.4**  **..R.....-...-..............**

**XD1.5**  **........-...-..............**

**XD1.7**  **........-...-..............**

**XDR10.2**  **........-...-..............**

**XDR10.3**  **........-...-..............**

**XDR10.9**  **........-...-G.............**

**XV3.2**  **........-...-..............**

**XV3.4**  **........-...-..............**

**LT576294**  **........-...-..............**

**21J3.1**  **........C...-.G............**

**21J3.2**  **........C...-.R............**

**21J3.5**  **........C...-.R............**

**21J3.6**  **........C...-..............**

**21J3.13**  **........C...-.G............**

**27G9.6**  **........C...-..............**

**27G9.8**  **........C...-..............**

**38V.15**  **........C...-..............**

**38V.18**  **........C...-..............**

**28C2**  **........C...-..............**

**28C3**  **........C...-..............**

**28C4**  **........C...-..............**

**LT576295**  **........C...A..............**

**LT576296**  **........C...-..............**

**Figure S2** Alignment of ITS-2 rDNA sequences of *Pharyngostrongylus kappa* and *Pharyngostrongylus patriciae* from different macropodid hosts. A dot indicates an identical nucleotide with respect to the sequence of XDR10.1; a dash indicates an insertion/deletion (indel) event. IUPAC codes indicate polymorphic positions in the sequences.
